# LitCovid, iSearch COVID-19 portfolio, and COVID-19 Global literature on coronavirus disease

**DOI:** 10.5195/jmla.2022.1274

**Published:** 2022-04-01

**Authors:** Laurel Scheinfeld

**Affiliations:** 1 laurel.scheinfeld@stonybrook.edu, Senior Assistant Librarian, Health Sciences Library, Stony Brook University, Stony Brook, NY

## Abstract

**LitCovid.** National Center for Biotechnology Information, US National Library of Medicine, 8600 Rockville Pike, Bethesda, MD 20894; https://www.ncbi.nlm.nih.gov/research/coronavirus/; free.

**iSearch COVID-19 portfolio.** Office of Portfolio Analysis, National Institutes of Health, 9000 Rockville Pike, Bethesda, MD 20892; https://icite.od.nih.gov/covid19/search/; free.

**COVID-19 Global literature on coronavirus disease.** World Health Organization, Avenue Appia 20, 1211 Geneva; https://search.bvsalud.org/global-literature-on-novel-coronavirus-2019-ncov/; free.

The SARS-CoV-2 pandemic created a unique situation in scholarly publishing: a sudden onset of published research on a topic and an extremely rapid rise in publishing activity. There has been a critical need to disseminate research findings quickly and efficiently, and many new tools have been developed to accomplish this. Databases created specifically for COVID-19 literature searching can provide a focused search experience for practitioners, researchers, librarians, and students. The National Institutes of Health (NIH) and the World Health Organization (WHO) have created several databases that are free and available via their websites, each with their own unique features. This review will cover LitCovid and iSearch COVID from NIH as well as the COVID-19 Global database from WHO.

LitCovid was created by computer scientists and bioinformationists at the National Center for Biotechnology Information (NCBI), a division of the National Library of Medicine (NLM) at NIH. LitCovid curates published research on COVID-19 from PubMed using a combination of automated search and human review and is updated daily. LitCovid supports keyword, Boolean, phrase, and PMID search entries. There is no advanced search option, but fields including title, abstract, journal, countries, authors, topics and e_drugs (chemicals) can be searched by typing the name of the field followed by a colon and the query (e.g., abstract: vaccine). There are eight broad topics (general information, mechanism, transmission, diagnosis, treatment, prevention, case report, and epidemic forecasting) that can be used in a field search. Search results can be sorted by recency or relevance. The five journals with the most published articles based on the search results are displayed and are available as filters. The five chemicals and countries mentioned most in the abstracts of resultant articles are also shown and can be used as filters. Additional features on the results page include a world map of countries mentioned in the abstracts and a histogram showing the number of published articles per week since February 2020, both based on the search results. The histogram can be used to filter the articles published in a particular week, but no other date filter is available. The entire set of results can be downloaded as a TSV or RIS file, and an RSS feed can be set up to receive ongoing results of the search. An individual article can be selected, bringing the user to a dedicated page with the article details and abstract, as well as a link to a full-text source and a list of “similar articles” that is curated based on topic and author-supplied keywords. There are also icons for sharing to social media. An individual article or selection of articles cannot be saved or exported from the LitCovid interface. User support consists of an About page and an FAQ page, and a link is provided for email inquiries and feedback. There is also a link to an article providing detailed descriptions of the methodology used to create and manage LitCovid [1]. The creators of LitCovid tested their method of curation and declared it superior with higher precision and better coverage than a search for COVID-19 in PubMed [1], but PubMed has since been updated with reportedly improved automatic term mapping (ATM) [2], and no newer comparison could be found. A search for the term COVID-19 brings a different number of results in PubMed compared to the total number of articles contained in LitCovid, illustrating that the methods used to curate articles for LitCovid differ from the ATM algorithm in PubMed (117,057 in PubMed versus 112,547 in LitCovid on March 26, 2021), but the quality of the two searches was not assessed.

The iSearch COVID-19 portfolio was developed by the Office of Portfolio Analysis, which is in the Office of the NIH Director. The main difference between this tool and LitCovid is that, in addition to curated COVID-19 content from PubMed, iSearch includes preprint server content from arXiv, bioRxiv, ChemRxiv, medRxiv, Preprints.org, Qeios, Research Square, and SSRN. As of April 19, 2021, there were 28,927 preprint articles. In addition, a public health focus is maintained by excluding articles that are not primarily about the science of COVID-19 but may be related to economics, education, and so on. The landing page for iSearch includes a search query box, a link to video tutorials, and a pictogram showing the number of articles added to the portfolio by source over the previous ten weeks. Many advanced searching options are available including Boolean, wildcard, and proximity searching, as well as two other advanced search features called “Fuzzy Search,” which accounts for alternate spellings and typos, and “Minimum Should Match,” which allows a searcher to specify a minimum number of terms that must be found. Advanced filters include publication date, publication source (PubMed or each preprint server), and publication type, which matches PubMed's publication types list. Each result links to a detailed record that can be customized to include selected fields. There is a permanent link available for each record, and a link to the source of the article includes the full text if available. Results can be exported to an Excel, RIS, or CSV file, or the URL of the search can be saved. A visualization tool is available in the results section that shows to what extent different subjects are represented in the titles and abstracts of the articles included in the search results. Artificial intelligence is used to analyze the titles and abstracts and generate the visualization, which is made up of different-sized clusters. An extensive user guide as well as three video tutorials are available and provide additional guidance on using advanced search techniques and refining results. A feedback form link goes directly to Outlook. For those who do not use Outlook, the email address to provide feedback or request additional help is available in the user guide. A submitted question was answered within a few hours.

Another database of COVID-19 literature is made available by WHO. The COVID-19 Global database includes international and multilingual literature and preprints from databases such as Global Index Medicus, Eurosurveillance, Airiti Library, Russian Science Index, Korean Science Citation Index, and Africa Wide Information, among many other sources. It is updated daily through automated and hand searches as well as expert-recommended articles. The complete list of sources and search strategies is provided in the help section of the database, which can be viewed in seven different languages and includes both a basic search box and an advanced search builder. The fields in the dropdown menu of the basic search are limited to title, abstract, author, or a combined search for title/abstract/subject; however, additional field codes can be used, a list of which is included in the Search Guide that is accessible from the homepage. Boolean, phrase, and truncation search techniques are supported. A wide array of filters are provided to focus a search including collection, subject, journal, database, date, study type, language, journal, clinical aspect, and full text availability. For each article in the results, a detailed record includes the citation, abstract, subjects, similar articles, and links to the article (if available) in full text, PubMed, and Google. Up to 50,000 citations can be exported in an RIS, CSV, or citation style format, up to 300 citations can be emailed, a page or a selection of citations can be printed, and an XML file can be generated. There is an RSS feed option, but the guide does not include instructions for setting this up, so additional help may be needed to utilize this function.

All three of these tools are free, easily accessible, updated frequently, highly user friendly for all levels of searchers, and provide useful instructional guides. Determining which to use or recommend will depend on the specific question or need of the user. LitCovid is a good starting point for general searches on COVID-19 literature. If preprints are needed, the iSearch database would be a more useful choice. And if international literature is desired, the WHO Global database is the better option.
